# An optimal combined slow-release nitrogen fertilizer and urea can enhance the decomposition rate of straw and the yield of maize by improving soil bacterial community and structure under full straw returning system

**DOI:** 10.3389/fmicb.2024.1358582

**Published:** 2024-06-19

**Authors:** Lihong Yu, Duo Li, Yifei Zhang, Yufeng Wang, Qin Yao, Kejun Yang

**Affiliations:** ^1^Heilongjiang Provincial Key Laboratory of Modern Agricultural Cultivation and Crop Germplasm, College of Agriculture, Heilongjiang Bayi Agricultural University, Daqing, China; ^2^Daqing Agricultural Technology Extension Center, Daqing, China

**Keywords:** maize, straw returning, slow-release N fertilizer, straw decomposition, bacterial diversity, microbial community structure

## Abstract

Under a full straw returning system, the relationship between soil bacterial community diversity and straw decomposition, yield, and the combined application of slow-release nitrogen and urea remains unclear. To evaluate these effects and provide an effective strategy for sustainable agricultural production, a 2-year field positioning trial was conducted using maize as the research object. Six experimental treatments were set up: straw returning + no nitrogen fertilizer (S1N0), straw returning + slow-release nitrogen fertilizer:urea = 0:100% (S1N1), straw returning + slow-release nitrogen fertilizer:urea = 30%:70% (S1N2), straw returning + slow-release nitrogen fertilizer:urea = 60%:40% (S1N3), straw returning + slow-release nitrogen fertilizer:urea = 90%:10% (S1N4), and straw removal + slow-release nitrogen fertilizer:urea = 30%:70% (S0N2). Significant differences (*p* < 0.05) were observed between treatments for Proteobacteria, Acidobacteriota, Myxococcota, and Actinobacteriota at the jointing stage; Proteobacteria, Acidobacteriota, Myxococcota, Bacteroidota, and Gemmatimonadota at the tasseling stage; and Bacteroidota, Firmicutes, Myxococcota, Methylomirabilota, and Proteobacteria at the maturity stage. The alpha diversity analysis of the soil bacterial community showed that the number of operational taxonomic units (OTUs) and the Chao1 index were higher in S1N2, S1N3, and S1N4 compared with S0N2 at each growth stage. Additionally, the alpha diversity measures were higher in S1N3 and S1N4 compared with S1N2. The beta diversity analysis of the soil bacterial community showed that the bacterial communities in S1N3 and S1N4 were more similar or closely clustered together, while S0N2 was further from all treatments across the three growth stages. The cumulative straw decomposition rate was tested for each treatment, and data showed that S1N3 (90.58%) had the highest decomposition rate. At the phylum level, straw decomposition was positively correlated with Proteobacteria, Actinobacteriota, Myxococcota, and Bacteroidota but significantly negatively correlated with Acidobacteriota. PICRUSt2 function prediction results show that the relative abundance of bacteria in soil samples from each treatment differed significantly. The maize yield of S1N3 was 15597.85 ± 1477.17 kg/hm^2^, which was 12.80 and 4.18% higher than that of S1N1 and S0N2, respectively. In conclusion, a combination of slow-release nitrogen fertilizer and urea can enhance the straw decomposition rate and maize yield by improving the soil bacterial community and structure within a full straw returning system.

## 1 Introduction

Crop straw is a significant by-product of agricultural production, and China is one of the leading straw producers globally. However, traditional straw disposal methods, such as leaving it in the field or burning it, can lead to severe soil quality degradation and environmental pollution. In addition, crop straw contains nutrients essential for crop growth (Henriksen and Breland, 2002), including carbon, nitrogen, phosphorus, and potassium. Therefore, returning straw to a field can have several positive effects. For instance, it improves soil physical properties and enhances soil organic matter content (Thompson et al., 2017; Duan et al., 2021). Additionally, it promotes soil nutrient cycling (Cao et al., 2022; Jayanthi and Gokila, 2023), regulates the structure of soil microbial communities (Bu et al., 2020; Wu et al., 2020; Yan et al., 2020), and ultimately reduces environmental pollution.

The carbon-to-nitrogen ratio (C/N) in straw is generally between 60 and 80. Unfortunately, returning large quantities of straw to a field can lead to a carbon and nitrogen imbalance, triggering a number of adverse effects. For instance, it can obstruct microorganism-based straw decomposition in the soil. Additionally, it can lead to a nitrogen imbalance in agricultural soil and a reduction in nitrogen utilization in crops. To address these issues, nitrogen fertilizer can be used to effectively regulate the C/N ratio after straw is returned to a field. This helps improve microbial activity (Zhang et al., 2022; Surigaoge et al., 2023), promote straw decomposition, and provide sufficient nitrogen fertilizer for crops. Previous studies have primarily focused on the effect of common urea fertilizer on straw decomposition (Liu et al., 2021; Mühlbachová et al., 2021). However, the high water solubility and fast nutrient conversion of urea fertilizer have led to inadequate nitrogen supplies in the later stages of straw decomposition and crop growth. To overcome these challenges, slow-release nitrogen fertilizers can be used. These fertilizers improve the nitrogen supply capacity of the soil and prevent the aforementioned problems by meeting the demands of straw decomposition and crop growth in terms of quantity, time, and space. Upon using slow-release nitrogen fertilizer with urea, the fertilization process can be simplified, and nitrogen fertilizer absorption and utilization in the middle and late plant growth stages can be improved. However, it is important to consider some potential issues associated with slow-release nitrogen fertilizers, such as increased costs, insufficient nitrogen supply, slow straw decomposition in the early stage, and delayed crop ripening. Taking these factors into account, slow-release nitrogen fertilizer with urea is a better choice to achieve increased crop yield.

Microorganisms serve as important connectors between the soil environment and plants. The structure, composition, and diversity of soil microorganisms are highly sensitive to environmental changes and can be used to assess soil quality and fertility (Jeffries et al., 2003; Luo et al., 2016). Soil microbial diversity and community structure directly or indirectly impact soil organic matter decomposition, nutrient cycling transformation, crop growth and development, and other significant factors. Research has demonstrated that straw returning alters the microorganisms’ habitat, directly or indirectly affecting their metabolic activities and changing their abundance and diversity (Gul et al., 2015). In a straw returning system, the diversity and abundance of soil bacterial and fungal community structures have been observed to change, with an overall improvement in diversity and abundance in the short term (Chen et al., 2019). Compared with conventional fertilizer systems, using slow-release fertilizers and straw returning can significantly increase the relative abundance of microorganisms (Zhang et al., 2021), enhance the diversity of bacterial and fungal communities, and promote soil carbon and nitrogen cycling (Feng et al., 2022).

A previous study report indicates that the long-term use of inorganic nitrogen fertilizers leads to a decrease in microbial diversity in soil (Zhang et al., 2022). Additionally, it increases the presence of pathogenic microorganisms in the soil and raises the risk of crop infection from soil-borne diseases (Gao et al., 2022). However, slow-release nitrogen fertilizers have shown the potential to enhance soil microbial or bacterial diversity and community structure compared with common urea (Teng et al., 2018; Liao et al., 2020). Therefore, it is crucial to investigate the alterations in soil bacterial communities under a straw returning system in order to enhance our understanding of maize microbial ecosystems.

Straw decomposition is a process of organic carbon mineralization and nutrient release controlled by soil microorganisms. All factors that affect microbial activity can affect straw decomposition. Studies have found that Actinobacteria, Firmicutes, and Proteobacteria play important roles in the corn straw decomposition process (Fan et al., 2014). Different microbial components show different decomposition functions for straw components. For example, Herzog et al. (2019) found that in the early stage of straw decomposition, the fast-cycling microorganisms Bacteroidetes and Helotiales dominate and are later replaced by Acidobacteria and Pleosporales. Therefore, the straw decomposition process is also an evolutionary process for the microbial community composition.

Currently, there are a limited number of studies investigating the impact of the combined application of conventional fertilizers and slow-release nitrogen fertilizers on soil bacterial diversity. Furthermore, no studies have examined the effects of different ratios between conventional and slow-release nitrogen fertilizers on soil bacterial diversity under straw returning conditions. Therefore, there is a need for further research in this area.

This study analyzes the soil bacterial community and straw decomposition rate data of maize soils with different ratios of slow-release nitrogen fertilizer and common urea under a full straw returning system. The analysis is conducted using Illumina MiSeq high-throughput sequencing technology. The aim is to explore the optimal application ratio for slow-release nitrogen and common urea in order to establish a theoretical basis and provide practical guidance for optimizing the soil microbial system and selecting the optimal fertilizer ratio.

## 2 Materials and methods

### 2.1 Experimental site

**Figure 1:**
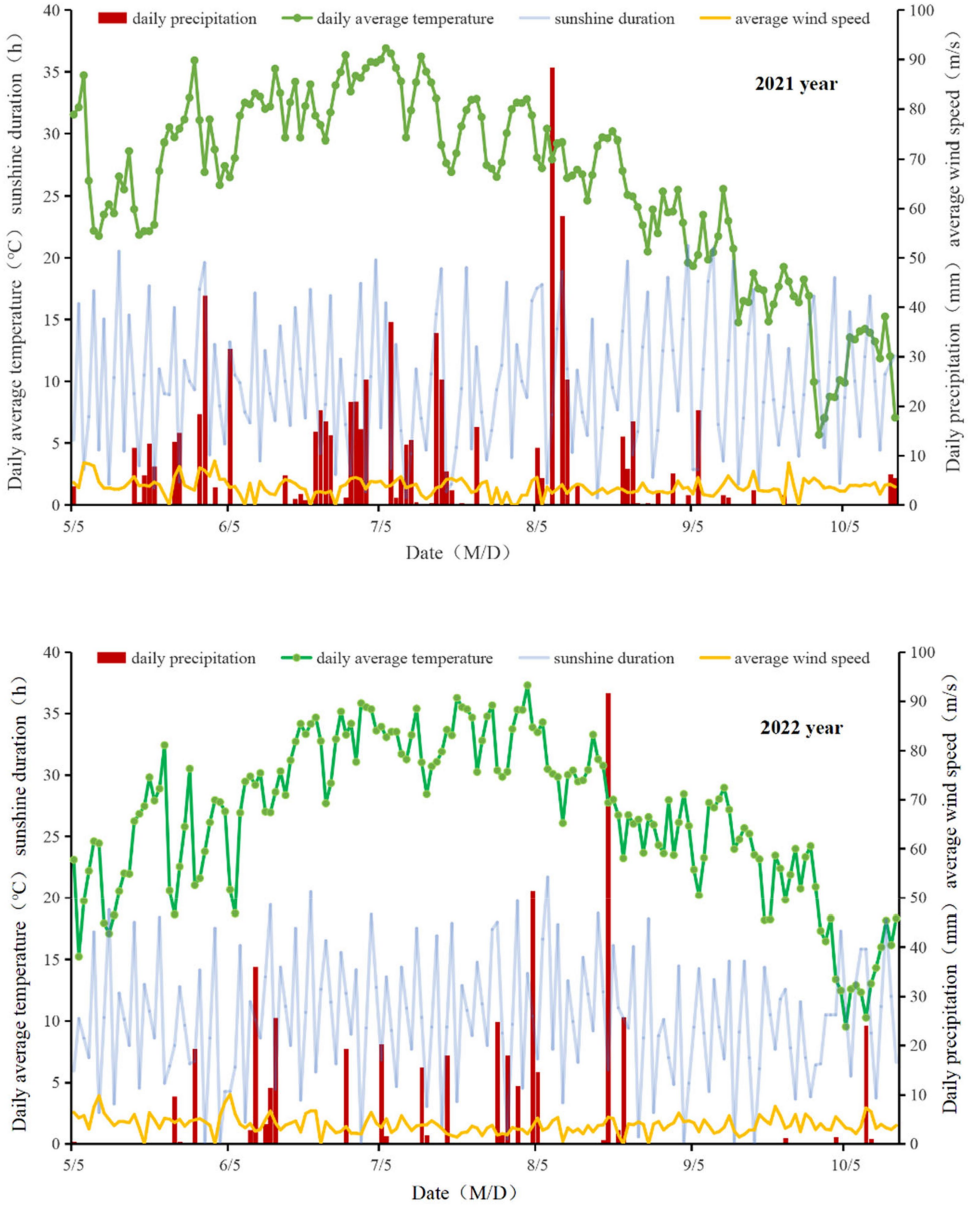
Daily average meteorological condition during maize growth stages in 2021 and 2022 year.

The experiment was conducted over a two-year period from 2021 to 2022 at the Anda Agricultural Science and Technology Park of Heilongjiang Bayi Agricultural University. It is located in Anda City, Heilongjiang Province, at coordinates 46.40°N and 125.34°E. The cropping system in the experimental plots is annual, with the previous crop being maize and the straw not being returned to the field. The climate in this area is classified as mid-temperate continental monsoon. During the 2021 growing season, the total rainfall was recorded at 766.2 mm, with an average temperature of 26.00°C, an average wind speed of 3.6 m/s, and a total of 1633.30 sunshine hours. In the following year, the 2022 growing season experienced a total rainfall of 470.00 mm, an average temperature of 25.33°C, an average wind speed of 4.06 m/s, and a total of 1848.71 sunshine hours (Figure 1).

### 2.2 Experimental design

The experiment was designed with two factors: straw treatment and nitrogen fertilizer treatment. The straw treatment included two options: (1) S1 for straw returning to the field and (2) S0 for straw not returning to the field. The nitrogen fertilizer treatment included five options: (1) N0 for no nitrogen fertilizer, (2) N1 for slow-release nitrogen fertilizer with urea at a ratio of 0:100%, (3) N2 for slow-release nitrogen fertilizer with urea at a ratio of 30:70%, (4) N3 for slow-release nitrogen fertilizer with urea at a ratio of 60:40%, and (5) N4 for slow-release nitrogen fertilizer with urea at a ratio of 90:10%. In total, there were six treatments: S1N0, S1N1, S1N2, S1N3, S1N4, and S0N2. The experiment used urea (N46%), slow-release fertilizer (N45%), monoammonium phosphate (P_2_O_5_ 50%, N10%), and potassium sulfate (K_2_O 50%) as fertilizers. Except for the S1N0 treatment, the total applications of P_2_O_5_, K_2_O, and N were the same in all other treatments, which were 75 kg/hm^2^, 52.5 kg/hm^2^, and 150 kg/hm^2^, respectively. All fertilizers were mixed according to the specified ratios and applied at once. The amount of fertilizer application, the process of fertilization, and the field management measures were the same in both years.

The experiment was conducted between 5 and 10 May in both 2021 and 2022. Maize seeds were planted during this period and the cobs were harvested between 10 and 15 October each year. After harvest, the maize straw was mechanically chopped and returned to the field. The straw was fully mulched and the dry weight of the returned straw was measured to be 6,000 kg/hm^2^. The experiment followed a randomized block design in 2021 and a positioning test in 2022. The specific variety of maize used was Xianyu 335, with a planting density of 67,000 plants per hectare. The area of each plot was 260 m^2^, with 8 rows, each 50 m in length and 0.65 m in width. Each treatment had three replications, resulting in a total of 18 plots.

The straw decomposition experiment was conducted using the nylon net bag method. A total of 94.5 g of straw (equivalent to the actual straw distribution in the field) was weighed and placed in a 100 mesh nylon net bag measuring 45 cm*35 cm. The placement of the nylon net bags in the field was synchronized with the soil tillage process.

### 2.3 Sample collection and determination

Samples of soil microbial diversity were collected from the root zone of plants. They were then transferred to 20 mL centrifuge tubes and frozen in liquid nitrogen. Subsequently, the samples were stored in a refrigerator at −80°C until they were tested.

Maize samples were collected at maturity. The measured production area consisted of only 156 m^2^ from the middle part of the middle 6 ridges. To ensure accuracy, one row on each side and a 5 m area on the front and back of each test area were excluded. For variety testing, 10 ears of corn were randomly and consecutively selected and brought to the laboratory. Several measurements such as moisture content, ear length, ear width, ear weight, number of rows per ear, number of kernels per row, and 100 grain weight were recorded. The yield of corn grain was then converted to 14% water content.

### 2.4 Soil DNA extraction, PCR amplification and Illumina MiSeq sequencing

Fifty-four samples of microbial genomic DNA were extracted using the E.Z.N.A.^®^ soil DNA Kit (Omega Bio-tek, Norcross, GA, United States), following the provided instructions. The quality and concentration of the DNA were assessed using 1.0% agarose gel electrophoresis and a NanoDrop^®^ ND-2000 spectrophotometer (Thermo Scientific Inc., United States). The DNA was then stored at −80°C until further use. The hypervariable region V3-V4 of the bacterial 16S rRNA gene was amplified using the primer pairs 338F (5′-ACTCCTACGGGAGGCAGCAG-3′) and 806R (5′-GGACTACHVGGGTWTCTAAT-3′) (Liu et al., 2016) with an ABI GeneAmp^®^ 9,700 PCR thermocycler (ABI, CA, United States). The PCR reaction mixture consisted of 4 μL of 5 × Fast Pfu buffer, 2 μL of 2.5 mM dNTPs, 0.8 μL of each primer (5 μM), 0.4 μL of Fast Pfu polymerase, 10 ng of template DNA, and ddH_2_O, with a final volume of 20 μL. The PCR amplification cycling conditions were as follows: initial denaturation at 95°C for 3 min, followed by 27 cycles of denaturation at 95°C for 30 s, annealing at 55°C for 30 s, and extension at 72°C for 45 s, with a final extension at 72°C for 10 min, and ending at 10°C. All samples were amplified in triplicate. The PCR product was extracted from a 2% agarose gel and purified using the AxyPrep DNA Gel Extraction Kit (Axygen Biosciences, Union City, CA, United States) according to the provided instructions. The quantification of the purified PCR product was performed using the Quantus™ Fluorometer (Promega, United States).

Following the standard protocols by Majorbio Bio-Pharm Technology Co., Ltd. (Shanghai, China), the purified amplicons were pooled in equimolar amounts and subjected to paired-end sequencing on an Illumina MiSeq PE300 platform (Illumina, San Diego, United States).

### 2.5 Data processing

The raw FASTQ files were de-multiplexed using an in-house Perl script. Subsequently, they underwent quality filtering with fastp version 0.19.6 (Chen et al., 2018). After this step, the files were merged using FLASH version 1.2.7 (Magoč and Salzberg, 2011) based on certain criteria, although not limited to: (i) truncating reads of 300 bp if the average quality score fell below 20 over a 50 bp sliding window. Any truncated reads shorter than 50 bp or containing ambiguous characters were discarded. (ii) Overlapping sequences were assembled if their length exceeded 10 bp, considering their overlapped sequence. (iii) The maximum allowed mismatch ratio in the overlap region was 0.2. Reads that could not be assembled were discarded. (iv) Samples were differentiated using barcodes and primers, with adjustments made to the sequence direction accordingly. Barcode matching required an exact match, while primer matching allowed for a maximum of 2 nucleotide mismatches. To classify the sequences into operational taxonomic units (OTUs), UPARSE 7.1 (Stackebrandt, 1994; Edgar, 2013) was utilized with a 97% similarity threshold. The representative sequence for each OTU was selected based on its abundance. Furthermore, the taxonomic classification of each representative sequence of an OTU was performed using the RDP Classifier version 2.2 (Wang et al., 2007) against the 16S rRNA gene database (e.g., Silva v138), with a confidence threshold set at 0.7.

### 2.6 Statistical analysis

Bioinformatic analysis of the soil was conducted using the Majorbio Cloud platform.^1^ Rarefaction curves and Alpha diversity indices were calculated to assess the microbial diversity, including observed OTUs, Chao1 richness, Shannon index, and Good’s coverage, using Mothur v1. 30.1 (Schloss et al., 2009). Principal coordinate analysis (PCoA) based on Bray–Curtis dissimilarity, using the Vegan v2.5–3 package, was employed to determine the similarity of microbial communities across samples. The PERMANOVA test, also using the Vegan v2.5–3 package, was used to evaluate the explanatory power of the treatment and its statistical significance in explaining the observed variation. To identify significantly abundant taxa at different taxonomic levels (phylum to genus), a linear discriminant analysis (LDA) effect size (LEfSe) (Segata et al., 2011) was performed.^2^ Additionally, the impact of soil physicochemical properties on bacterial community structure was examined using db-RDA analysis with Vegan v2.5–3 software. The axes’ values and arrow lengths in the analysis represented the significance of each property in shaping the distribution of taxa in the communities. Linear regression analysis was used to establish the correlation between the prominent physicochemical features identified by the db-RDA analysis and the Alpha diversity indices of the microorganisms.

The data in this study were collected in 2022. Data analysis was conducted using Excel 2019 (Microsoft,United Stated) and SAS 9.4 software. Multiple comparisons were performed using one-way analysis of variance and Duncan’s procedure, while correlations were analyzed using Pearson’s procedure. Graphical representations were created using the Majorbio Cloud platform (see text footnote 1), Origin 2021 (Originlab, OriginPro 2021, United Stated).

## 3 Results

### 3.1 Soil bacterial community composition

In this experiment, a total of six treatments with three replicates for each treatment were analyzed using Illumina MiSeq high-throughput sequencing technology. A total of 54 samples at the jointing, tasseling, and maturity stages were included in the analysis. Data regarding soil microbial diversity were sampled and leveled before statistical analysis, resulting in a total of 1,613,574 valid sequences. The representative sequences of each operational taxonomic unit (OTU) were used to classify the taxonomic composition of the soil microbial community at the phylum, order, and species levels. Overall, 43 bacterial phyla, 147 bacterial classes, 369 bacterial orders, 582 bacterial families, 1,092 bacterial genera, 2,464 bacterial species, and 8,497 bacterial OTUs were detected across all stages. The top 10 most abundant bacterial communities were classified as the dominant group, while the remaining were classified as others.

#### 3.1.1 Soil bacteria composition at the phylum level

**Figure 2:**
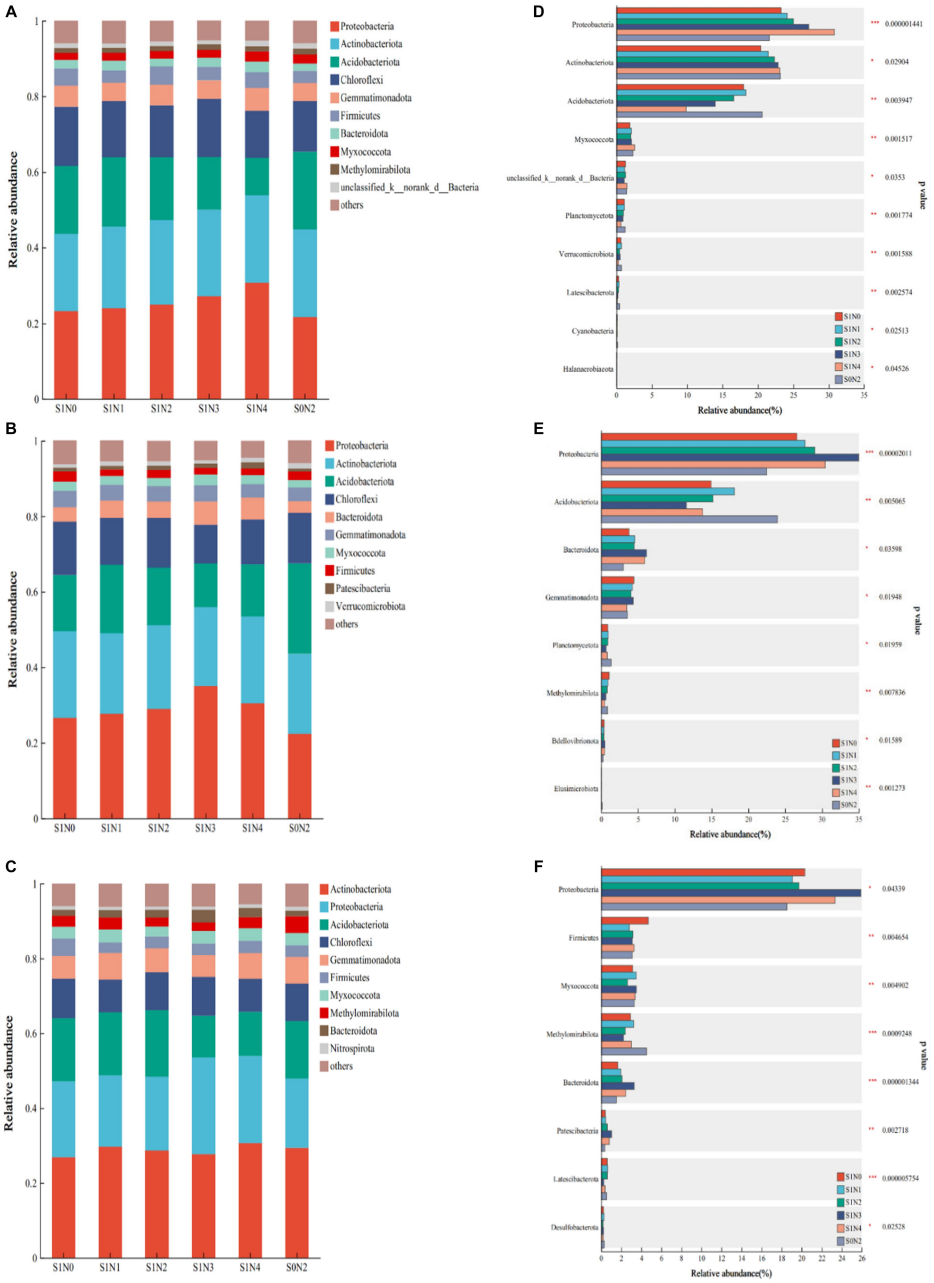
Bacterial flora composition and difference test at the phylum level. **(A)** Jointing phylum composition, **(B)** tasseling phylum composition, **(C)** maturity phylum composition, **(D)** test for difference in jointing phylum, **(E)** test for difference in tasseling phylum, **(F)** test for difference in maturity phylum. *, **, *** significant at *p* < 0.05, *p* < 0.01, and *p* < 0.001, respectively, the same in Figures 3, 4.

At the jointing, tasseling, and maturity stages, 42, 41, and 43 bacterial phyla were detected, respectively. The dominant bacterial phyla remained consistent across all treatments at these stages. The top 10 phyla at each fertility period are shown in Figures 2A–C, ranked by relative abundance from high to low. Eight common dominant phyla were consistently observed in each fertility period: Proteobacteria (21.12–39.23%), Actinobacteriota (16.77–29.53%), Acidobacteriota (7.07–26.66%), Chloroflexi (4.24–19.78%), Gemmatimonadota (2.08–8.71%), Firmicutes (0.96–5.76%), Bacteroidota (1.48–8.90%), and Myxococcota (1.58–4.18%). The differential phyla observed were Methylomirabilota (0.96–2.7%) at the jointing stage, Patescibacteria (0.54–1.99%) at the tasseling stage, and Verrucomicrobiota (0.49–5.18%) and Methylomirabilota (0.98–4.75%), and Nitrospirota (0.54–1.38%) at the maturity stage.

Figures 2D–F show the top 10 bacterial phyla with significant differences, and the significantly different phyla differed among the growth stages. At the jointing stage, Proteobacteria, Acidobacteriota, and Myxococcota phyla had extremely significant differences (*p* < 0.01), and Actinobacteriota phyla had significant differences (*p* < 0.05). At the tasseling stage, Proteobacteria, Acidobacteriota, and Myxococcota phyla had extremely significant differences (*p* < 0.01), and Bacteroidota and Gemmatimonadota phyla had significant differences (*p* < 0.05). At the maturity stage, Bacteroidota, Firmicutes, Myxococcota, and Methylomirabilota had extremely significant differences (*p* < 0.01), and Proteobacteria had a significant difference (*p* < 0.05).

#### 3.1.2 Soil bacteria composition at the class level

**Figure 3:**
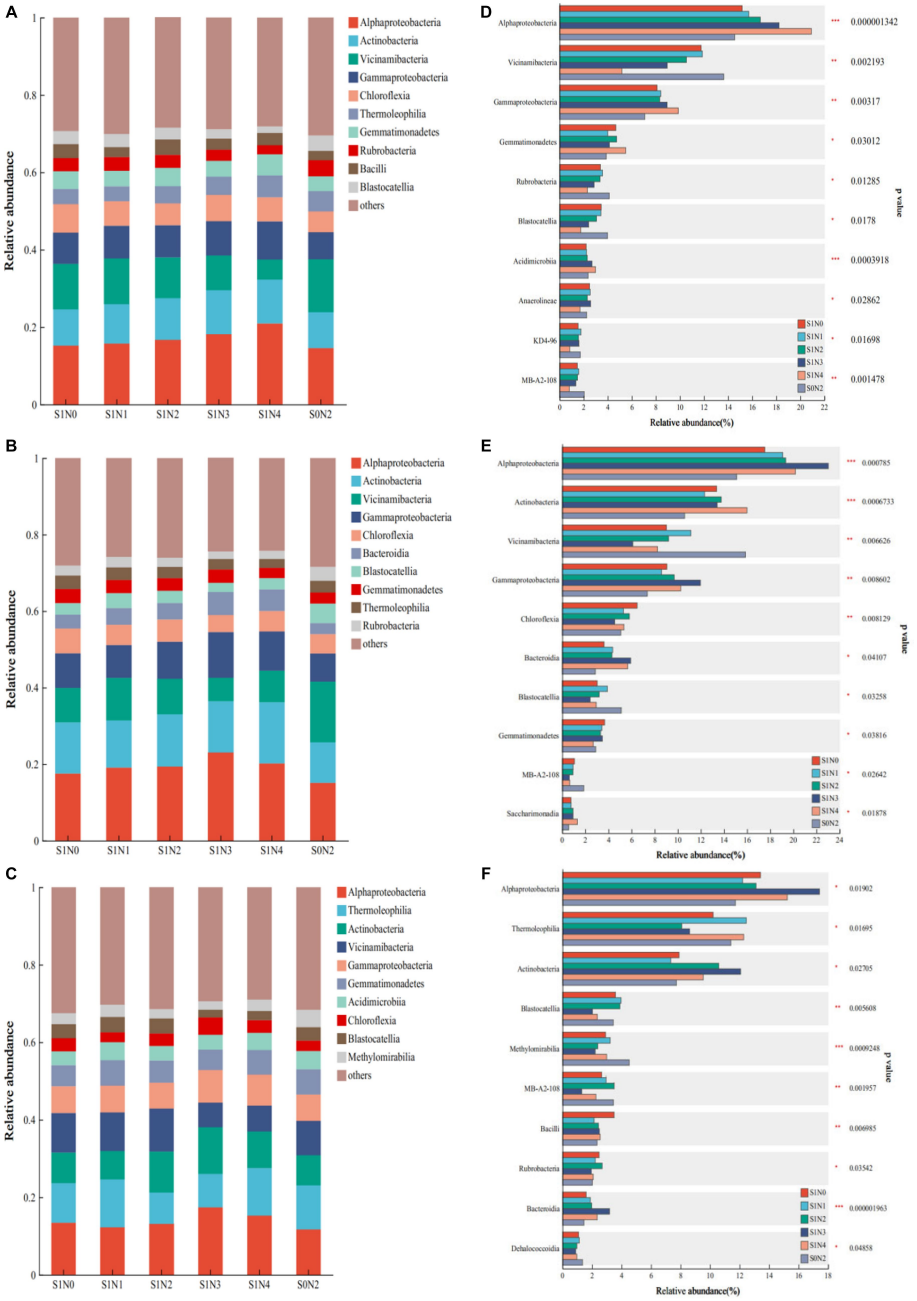
Bacterial flora composition and difference test at the class level. **(A)** Jointing class composition, **(B)** tasseling class composition, **(C)** maturity class composition, **(D)** test for difference in jointing class, **(E)** test for difference in tasseling class, **(F)** test for difference in maturity class.

Figure 3 shows the presence of seven common dominant classes during each reproductive period. These classes are Alphaproteobacteria, Actinobacteria, Vicinamibacteria, Gammaproteobacteria, Chloroflexia, Gemmatimonadetes, and Thermoleophilia. Additionally, a differential comparison was conducted at different stages. At the jointing stage, there were extremely significant differences (*p* < 0.01) observed in the abundances of Alphaproteobacteria, Vicinamibacteria, and Gammaproteobacteria. Similarly, at the tasseling stage, there were extremely significant differences observed in the abundances of Alphaproteobacteria, Actinobacteria, Vicinamibacteria, Gammaproteobacteria, and Chloroflexia. Finally, at the maturity stage, Blastocatellia and Methylomirabilia showed extremely significant differences.

#### 3.1.3 Soil bacteria composition at the genus level

**Figure 4:**
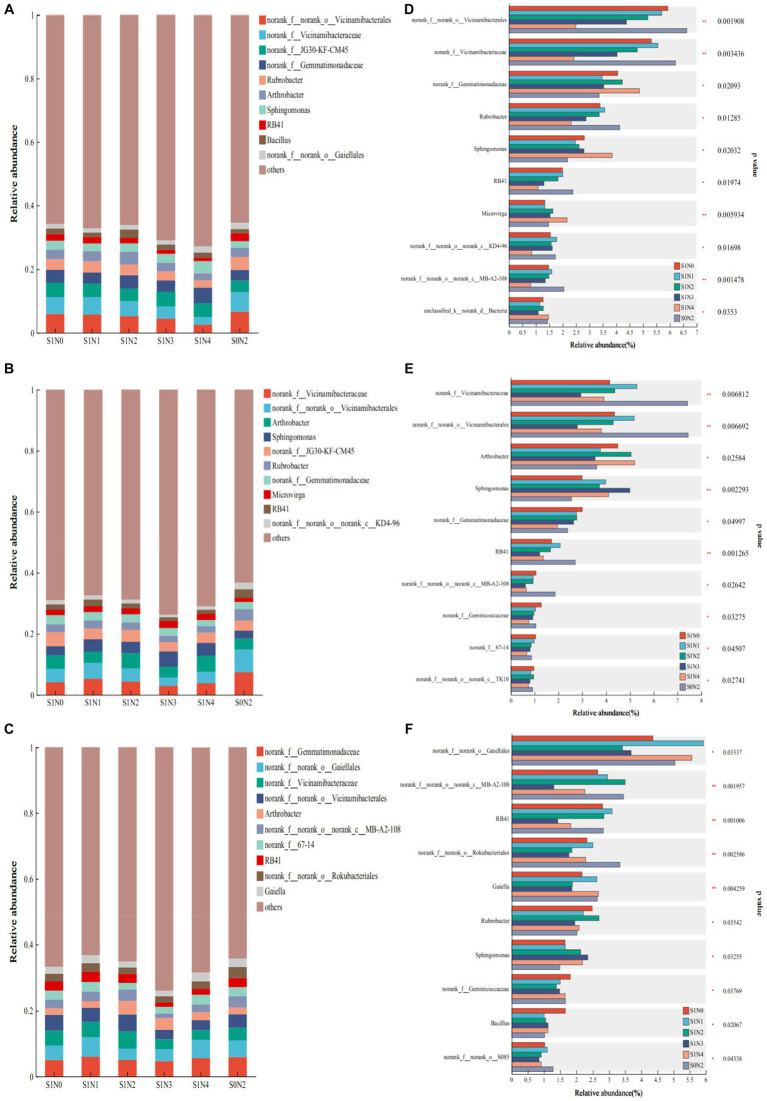
Bacterial flora composition and difference test at the genus level. **(A)** Jointing genus composition, **(B)** tasseling genus composition, **(C)** maturity genus composition, **(D)** test for difference in jointing genus, **(E)** test for difference in tasseling genus, **(F)** test for difference in maturity genus.

Figures 4A–C displays the dominant bacterial genera during each growth period, while Figures 4D–F highlights the bacterial genera with significant differences. Among the 10 dominant bacterial genera in the jointing stage, six genera exhibited significant differences. Notably, norank_f__norank_o__Vicinamibacterales and norank_f__Vicinamibacteraceae displayed extremely significant differences (*P <* 0.01), while norank_f__Gemmatimonadaceae, Rubrobacter, Sphingomonas, and RB41 showed significant differences (*P <* 0.05). During the tasseling period, there were six dominant bacterial genera with significant differences. Specifically, norank_f__Vicinamibacteraceae, norank_f__norank_o__Vicinamibacterales, and Sphingomonas demonstrated extremely significant differences (*P* < 0.01), while Arthrobacter, norank_f__Gemmatimonadaceae, and RB41 exhibited significant differences (*P* < 0.05). In the maturity stage, there were five dominant bacterial genera with significant differences. Remarkably, RB41, norank_f__norank_o__Rokubacteriales, and Gaiella displayed extremely significant differences (*P* < 0.01), while norank_f__norank_o__Gaiellales and norank_f__norank_o__norank_c__MB-A2-108 showed significant differences (*P* < 0.05).

### 3.2 Soil bacterial community alpha diversity analysis

Alpha diversity was assessed upon analyzing the overall horizontal coverage of the bacterial community, as well as the number of OTUs, ACE index, Chao1 index, Simpson index, and Shannon index. The coverage rates for each treatment during different growth stages exceeded 95%, indicating that the sequencing was sufficient to reflect the bacterial communities in the soil samples accurately. Table 1 reveals that the number of OTUs and the Chao1 index for S1N2, S1N3, and S1N4 were higher than S0N2 at each growth stage. Additionally, S1N3 and S1N4 had higher values than S1N2. However, no clear patterns were observed between S1N1 and S0N2. The ACE index for S1N4 was the highest among all growth stages, and there was no significant difference between S1N3 and S1N4. However, there was no obvious relationship between S1N1 and S0N2. The Shannon index for S1N3 and S1N4 was higher than S0N2, but there was no significant difference between S1N3 and S1N4. Overall, when straw is returned to a field, the combined application of slow-release nitrogen fertilizer and urea can enhance the soil bacterial community alpha diversity. Furthermore, a higher ratio of slow-release fertilizer appears to have a positive impact on soil bacterial diversity.

**Table 1:** Alpha diversity index at different growth stages.

Stage	Treatment	Coverage	Number of OTUs	ACE index	Chao1 index	Simpson index	Shannon index
Jointing	S1N0	96.17%	3025.33 ± 53.98c	4644.29 ± 382.13cd	4357.93 ± 98.33cd	0.004277 ± 0.0004306ab	6.60 ± 0.03b
	S1N1	96.02%	3106.33 ± 50.36ab	4815.45 ± 411.71bc	4493.39 ± 78.53bc	0.003650 ± 0.0000684b	6.66 ± 0.02ab
	S1N2	96.17%	2933.00 ± 58.97d	5141.34 ± 165.23ab	4322.25 ± 113.25cd	0.004962 ± 0.0007579a	6.53 ± 0.02c
	S1N3	95.90%	3075.67 ± 29.19bc	5376.61 ± 144.48a	4594.22 ± 137.33ab	0.004232 ± 0.0001344ab	6.62 ± 0.04ab
	S1N4	95.82%	3182.00 ± 4.36a	5583.35 ± 112.29a	4722.30 ± 104.90a	0.004189 ± 0.0001907ab	6.68 ± 0.04a
	S0N2	96.31%	2909.33 ± 37.00d	4254.63 ± 116.63d	4241.93 ± 155.57d	0.004271 ± 0.0005448ab	6.54 ± 0.05c
Tasseling	S1N0	96.00%	3129.33 ± 16.44b	4867.90 ± 547.28a	4528.05 ± 80.39a	0.005450 ± 0.0009091a	6.62 ± 0.02c
	S1N1	95.98%	3172.67 ± 135.15ab	4876.29 ± 668.19a	4561.31 ± 342.13a	0.005634 ± 0.0011728a	6.64 ± 0.01bc
	S1N2	95.81%	3260.33 ± 126.34ab	5270.73 ± 433.99a	4780.29 ± 184.24a	0.005452 ± 0.0008270a	6.66 ± 0.03b
	S1N3	95.73%	3358.33 ± 85.98a	4893.00 ± 160.82a	4796.83 ± 186.31a	0.004133 ± 0.0003605a	6.76 ± 0.02a
	S1N4	95.77%	3300.00 ± 106.78ab	5364.17 ± 550.77a	4794.07 ± 222.82a	0.004395 ± 0.0009230a	6.73 ± 0.01a
	S0N2	95.95%	3182.67 ± 121.69ab	4916.21 ± 628.83a	4559.04 ± 233.00a	0.004287 ± 0.0006818a	6.67 ± 0.03b
Maturity	S1N0	96.22%	2988.33 ± 176.10bc	4334.60 ± 253.25c	4292.72 ± 124.96b	0.004348 ± 0.0007636a	6.55 ± 0.13ab
	S1N1	96.27%	2937.33 ± 49.24c	4265.79 ± 110.15c	4205.57 ± 133.70b	0.004453 ± 0.0005141a	6.51 ± 0.03ab
	S1N2	96.20%	2984.67 ± 126.98bc	4336.48 ± 187.67c	4316.64 ± 133.09b	0.004334 ± 0.0003621a	6.53 ± 0.09ab
	S1N3	95.57%	3227.33 ± 77.86ab	5728.99 ± 218.42a	4821.52 ± 195.69a	0.005969 ± 0.0038291a	6.59 ± 0.10ab
	S1N4	95.60%	3259.00 ± 96.02a	5845.98 ± 142.28a	4947.24 ± 83.79a	0.004732 ± 0.0012070a	6.64 ± 0.03a
	S0N2	96.12%	2869.67 ± 195.98c	5187.51 ± 184.02b	4306.44 ± 208.93b	0.005516 ± 0.0013942a	6.38 ± 0.19b
	Stage (S)	**	**	NS	**	NS	**
	Treatment (T)	**	**	**	**	NS	**
	(S*T)	**	**	**	**	**	**

Values are mean ± standard deviation, lowercase letters in the same column indicate significant difference among treatments (*P* < 0.05). NS, no signifcant; ** Signifcant at *p* < 0.01.

### 3.3 Soil bacterial community beta diversity analysis

**Figure 5:**
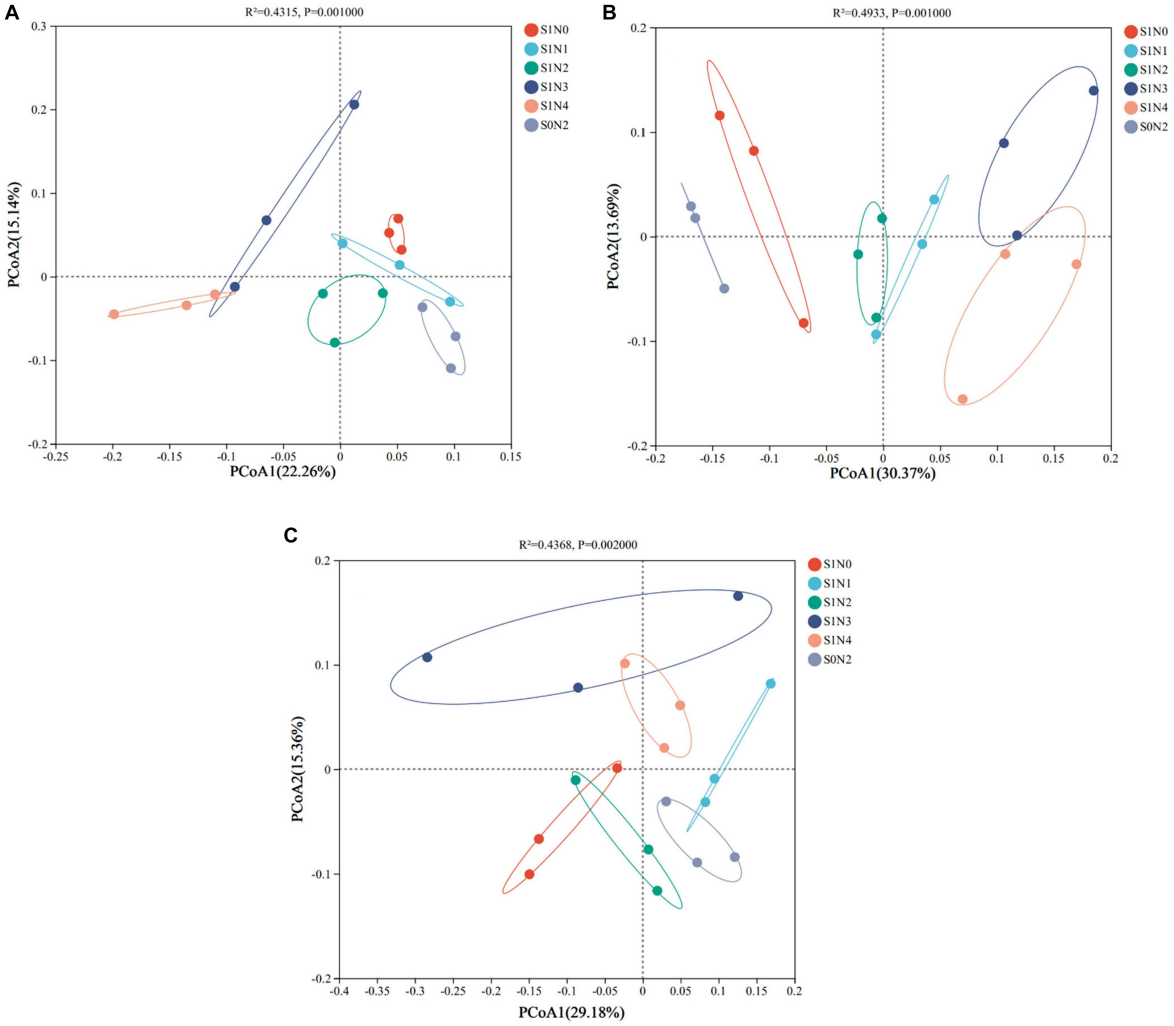
Principal coordinate analysis of soil bacterial OTUs (PCoA). **(A)** Jointing stages, **(B)** tasseling stages, **(C)** maturity stages.

Based on the abundance of OTUs, the principal coordinate analysis (PCoA) results are presented in Figure 5. The soil samples from the three growth stages formed distinct clusters along both the horizontal and vertical axes. In the jointing stage, the horizontal axis accounted for 22.26% of the total sample variation, while the vertical axis contributed 15.14%, collectively explaining 37.40% of the variation in the soil bacterial community. The PERMANOVA test (*R*^2^ = 0.4315, *p* = 0.001) indicated significant differences in the bacterial communities among the different treatments. Specifically, the S1N3 and S1N4 treatments were mainly located in the second and third quadrants, far from the other treatments. During the tasseling stage, the horizontal and vertical axes explained 30.37 and 13.69% of the community variation, respectively, accounting for a total of 44.06% of the variation in the soil bacterial community. The PERMANOVA test (*R*^2^ = 0.4933, *p* = 0.001) revealed that S1N3 and S1N4 clustered close to the right, while S1N1 and S1N2 were clustered around the origin position. This suggests that the bacterial communities of S1N3 and S1N4, as well as S1N1 and S1N2, had similar compositions, while S0N2 was located to the left of the coordinate system. In the maturity stage, the horizontal and vertical axes explained 29.18 and 15.36% of the community variation, respectively, contributing to a total of 44.54% of the variation in the soil bacterial community. The PERMANOVA test (*R*^2^ = 0.4368, *p* = 0.002) supported these findings. The beta diversity analysis of the soil bacterial community revealed that the bacterial communities in S1N3 and S1N4 were closely associated. At the same time, S0N2 showed dispersion and was distant from all treatments across the three growth stages.

### 3.4 Straw decomposition characteristics

**Figure 6:**
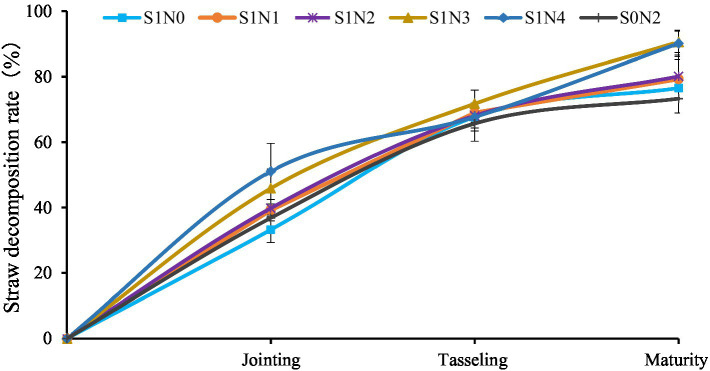
The dynamic of maize straw cumulative decomposition rate.

The decomposition rule of maize straw in different treatments throughout the whole process was consistent in that the decomposition speed was faster in the early stage (before tasseling) than in the later stages (after tasseling) (Figure 6). At the jointing stage, the straw decomposition rate in each treatment was sorted as S1N4 (51.07%) > S1N3 (45.85%) > S1N2 (39.79%) > S1N1 (39.22%) > S0N2 (36.81%) > S1N0 (33.22%). At the tasseling stage, the cumulative decomposition straw rate was sorted as S1N3 (71.73%)>S1N1 (68.97%)>S1N2 (68.09%)>S1N4 (67.70%)>S1N0 (67.67%)>S0N2 (65.67%). At the maturity stage, the order of cumulative straw decomposition rate was sorted as S1N3 (90.58%) > S1N4 (90.14%) > S1N2 (80.10%) > S1N1 (79.26%) > S1N0 (76.52%) > S1N0 (73.29%). At the maturity stage, The cumulative straw decomposition rate in S1N3 and S1N4 were top level, which differed significantly (*P* < 0.05) from other treatments.

### 3.5 Correlation between straw decomposition rate and soil bacterial community

#### 3.5.1 Correlation between straw decomposition rate and dominant phylum

**Figure 7:**
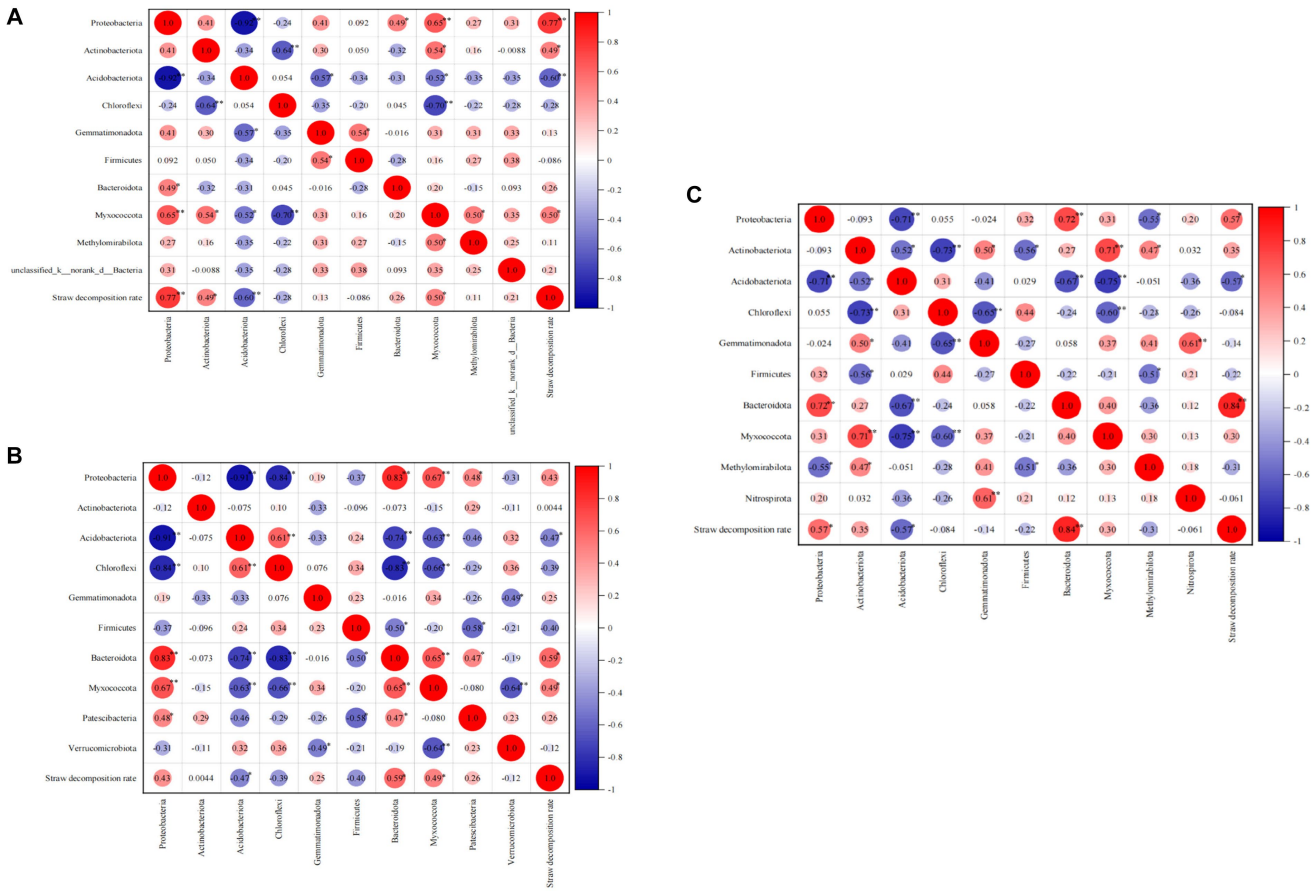
Correlation analysis of straw decomposition rate and dominant soil bacteria at the phylum level. The number indicate correlation coefficient, the same below. **(A)** Jointing stages, **(B)** tasseling stages, **(C)** maturity stages.

Correlation analyses were conducted at both the phylum and class levels to investigate the impact of predominant bacterial groups on the straw decomposition rate (Figure 7). At the jointing stage, Proteobacteria (correlation coefficient, 0.77; *p* < 0.01), Actinobacteriota (0.49, *p* < 0.05), Myxococcota (0.50, *p* < 0.05), and Bacteroidota (0.26) showed positive correlations with the straw decomposition rate. In contrast, Acidobacteriota (−0.60, *p* < 0.01) and Chloroflexi (−0.28) showed negative correlations with the straw decomposition rate. At the tasseling stage, Proteobacteria (0.43), Bacteroidota (0.59, *p* < 0.05), and Myxococcota (0.49, *p* < 0.05) exhibited positive correlations with the straw decomposition rate. In contrast, Acidobacteriota (−0.47, *p* < 0.05) and Chloroflexi (−0.28) showed negative correlations with the straw decomposition rate. At the maturity stage, Proteobacteria (0.43, *p* < 0.05), Bacteroidota (0.84, *p* < 0.01), Actinobacteriota (0.35), and Myxococcota (0.30) displayed positive correlations with the straw decomposition rate. In contrast, Acidobacteriota (−0.57, *p* < 0.05) showed a negative correlation with the straw decomposition rate. In summary, Proteobacteria, Actinobacteriota, Myxococcota, and Bacteroidota exhibited positive correlations with straw decomposition at the phylum level, indicating their favorable effect on straw decomposition. On the other hand, Acidobacteriota and Chloroflexi showed significant negative correlations with straw decomposition, suggesting their unfavorable effect on straw decomposition.

Figure 7 demonstrates significant correlations between bacterial phyla. During the jointing stage, the relative abundance of Proteobacteria showed significant positive correlations with Myxococcota and Bacteroidota but a significant negative correlation with Acidobacteriota. Additionally, negative correlations were observed between Chloroflexi, Actinobacteriota, Gemmatimonadota, Myxococcota, and Acidobacteriota. During the tasseling stage, the relative abundance of Myxococcota exhibited significant positive correlations with Proteobacteria and Bacteroidota but significant negative correlations with Acidobacteriota, Chloroflexi, and Verrucomicrobiota. Last, at the maturity stage, the relative abundance of Bacteroidota displayed a positive correlation with Proteobacteria but a significant negative correlation with Acidobacteriota. These findings highlight the presence of a complex, competitive relationship among bacteria.

#### 3.5.2 Correlation between straw decomposition rate and dominant class

**Figure 8:**
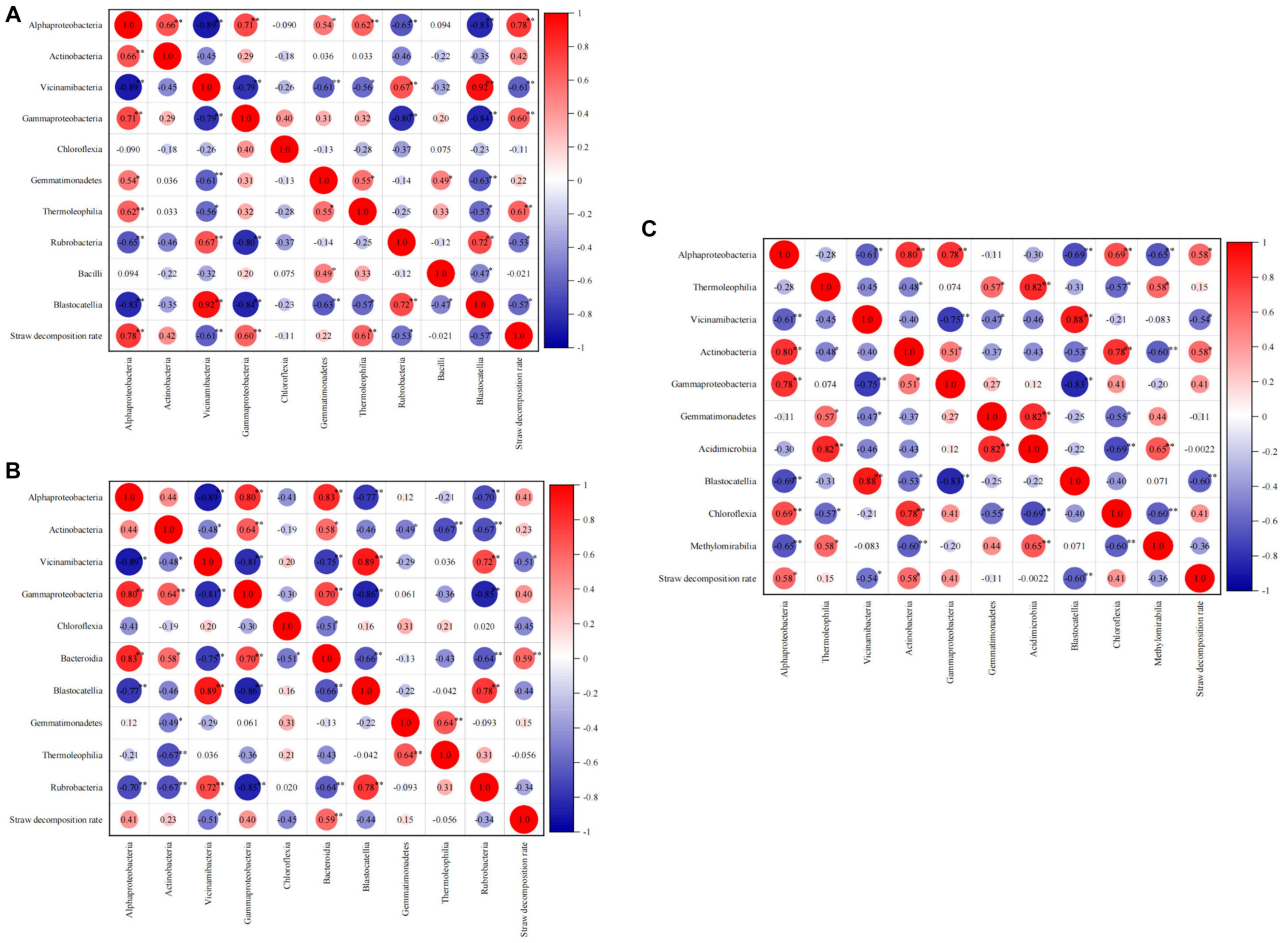
Correlation analysis of straw decomposition rate and dominant soil bacteria at the class level.

As shown in Figure 8, during the jointing stage, Alphaproteobacteria, Gammaproteobacteria, and Thermoleophilia exhibited significant positive correlations (*p* < 0.01) with the straw decomposition rate. The correlation coefficients for Alphaproteobacteria, Gammaproteobacteria, and Thermoleophilia were 0.78, 0.60, and 0.61, respectively. On the other hand, Vicinamibacteria (−0.61, *p* < 0.01) and Rubrobacteria (−0.53, *p* < 0.05) showed negative correlations with straw decomposition. Moving on to the tasseling stage, Bacteroidia (0.59, *p* < 0.01) and Alphaproteobacteria (0.41) were positively correlated with the straw decomposition rate, while Vicinamibacteria (−0.51, *p* < 0.05) exhibited a significant negative correlation. At the maturity stage, Alphaproteobacteria and Actinobacteria were positively correlated with straw decomposition, whereas Vicinamibacteria (−0.54, *p* < 0.05) and Blastocatellia (−0.60, *p* < 0.01) showed negative correlations. In summary, Alphaproteobacteria and Vicinamibacteria were identified as the two most influential bacterial classes affecting straw decomposition throughout all stages. Specifically, Alphaproteobacteria exhibited a positive correlation with the straw decomposition rate, while Vicinamibacteria showed a negative correlation.

#### 3.5.3 Correlation between straw decomposition rate and dominant genera

**Figure 9:**
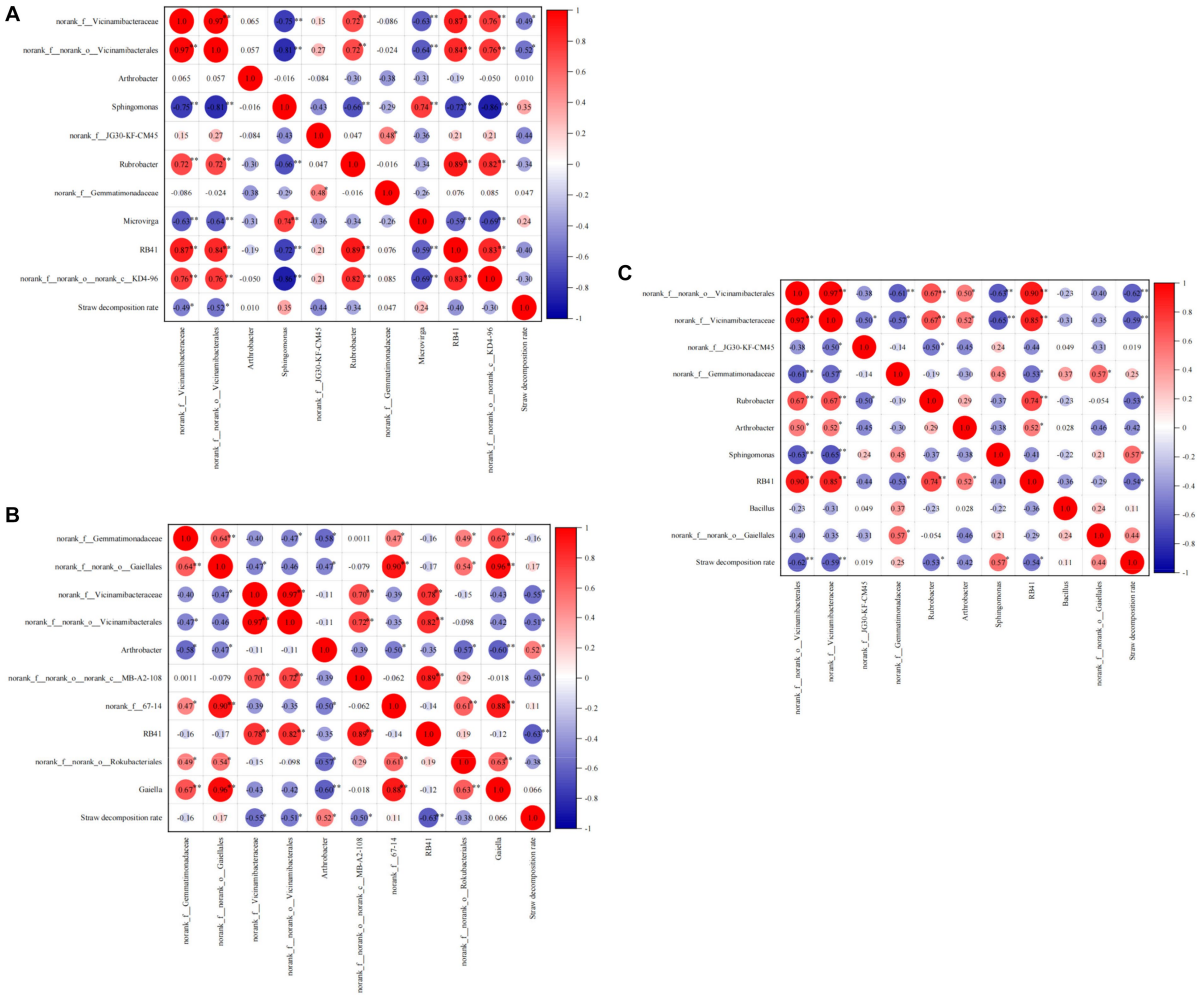
Correlation analysis of straw decomposition rate and dominant soil bacteria at the genus level. **(A)** Jointing stages, **(B)** tasseling stages, **(C)** maturity stages.

During the jointing stage, there was a significant negative correlation between norank_f__norank_o__Vicinamibacterales (−0.62, *p* < 0.01) and norank_f__Vicinamibacteraceae (−0.59, *p* < 0.01) with the straw decomposition rate. On the other hand, Vicinamibacteria (0.57, *p* < 0.05) showed a positive correlation with straw decomposition. At the tasseling stage, norank_f__Vicinamibacteraceae (−0.49, *p* < 0.05) and norank_f__norank_o__Vicinamibacterales (−0.52, *p* < 0.05) were negatively correlated with the straw decomposition rate. Finally, at the maturity stage, Arthrobacter (0.52, *p* < 0.05) showed a positive correlation with straw decomposition, while RB41 (−0.68, *p* < 0.01) exhibited a negative correlation (Figure 9).

### 3.6 PICRUSt function prediction analysis of soil bacterial communities

PICRUSt2 was used to perform functional prediction analysis on bacterial 16 s amplicon sequencing data (Tables 2–4). The results showed that among the top 20 functional genes with high relative abundance in the secondary functional layer of the KEGG database, the relative abundances of bacteria in soil samples from each treatment differed significantly. Among them, S1N3 and S1N4 significantly increased the relative abundance of carbohydrate metabolism, amino acid metabolism, membrane transport, cellular community-prokaryotes, signal transduction, lipid metabolism, xenobiotics biodegradation, and metabolism in the three growth stages; significantly reduced the relative abundances of the global and overview maps, metabolism of cofactors and vitamins, translation, nucleotide metabolism, biosynthesis of other secondary metabolites, folding, sorting, and degradation.

**Table 2:** Functions of bacterial communities at jointing stage (%).

Functions	S1N0	S1N1	S1N2	S1N3	S1N4	S0N2
1	40.82 ± 0.04ab	40.81 ± 0.04ab	40.73 ± 0.05b	40.58 ± 0.14c	40.34 ± 0.08d	40.94 ± 0.10a
2	9.33 ± 0.03a	9.33 ± 0.07a	9.36 ± 0.05a	9.26 ± 0.11a	9.13 ± 0.01b	9.36 ± 0.09a
3	8.14 ± 0.02b	8.15 ± 0.03b	8.17 ± 0.02ab	8.19 ± 0.04a	8.14 ± 0.00b	8.14 ± 0.03ab
4	4.49 ± 0.01a	4.48 ± 0.02a	4.46 ± 0.02b	4.45 ± 0.02b	4.44 ± 0.01b	4.50 ± 0.01a
5	4.25 ± 0.01a	4.25 ± 0.01ab	4.23 ± 0.01abc	4.22 ± 0.02c	4.23 ± 0.01bc	4.26 ± 0.00a
6	2.92 ± 0.02a	2.91 ± 0.02ab	2.86 ± 0.02bc	2.83 ± 0.05cd	2.80 ± 0.03d	2.95 ± 0.02a
7	2.71 ± 0.04cd	2.72 ± 0.01cd	2.78 ± 0.03bc	2.84 ± 0.09ab	2.88 ± 0.06a	2.66 ± 0.05d
8	2.41 ± 0.01a	2.41 ± 0.02a	2.39 ± 0.01a	2.38 ± 0.03a	2.38 ± 0.02a	2.41 ± 0.02a
9	2.34 ± 0.01ab	2.34 ± 0.01bc	2.33 ± 0.01c	2.31 ± 0.01d	2.30 ± 0.01d	2.35 ± 0.01a
10	2.30 ± 0.03c	2.30 ± 0.03c	2.32 ± 0.02bc	2.37 ± 0.07ab	2.41 ± 0.04b	2.27 ± 0.04c
11	2.24 ± 0.02bc	2.23 ± 0.02c	2.27 ± 0.01bc	2.27 ± 0.03b	2.34 ± 0.03a	2.20 ± 0.01d
12	2.20 ± 0.01c	2.21 ± 0.02bc	2.22 ± 0.01abc	2.23 ± 0.02a	2.22 ± 0.01ab	2.21 ± 0.00c
13	1.86 ± 0.02c	1.88 ± 0.03c	1.92 ± 0.02b	1.97 ± 0.05a	1.97 ± 0.02a	1.86 ± 0.00c
14	1.56 ± 0.01ab	1.56 ± 0.01ab	1.55 ± 0.01c	1.55 ± 0.01bc	1.55 ± 0.01c	1.57 ± 0.00a
15	1.56 ± 0.01ab	1.57 ± 0.01ab	1.57 ± 0.00ab	1.58 ± 0.01a	1.58 ± 0.00a	1.56 ± 0.02b
16	1.41 ± 0.01a	1.40 ± 0.01ab	1.39 ± 0.01b	1.37 ± 0.02c	1.36 ± 0.01c	1.41 ± 0.01a
17	1.20 ± 0.01a	1.20 ± 0.01a	1.17 ± 0.01b	1.16 ± 0.03bc	1.15 ± 0.01c	1.21 ± 0.01a
18	1.10 ± 0.00a	1.10 ± 0.01a	1.09 ± 0.01a	1.11 ± 0.01a	1.11 ± 0.00a	1.10 ± 0.02a
19	0.85 ± 0.01c	0.85 ± 0.01bc	0.86 ± 0.01c	0.87 ± 0.01b	0.89 ± 0.00a	0.85 ± 0.01c
20	0.79 ± 0.03bc	0.77 ± 0.03bc	0.80 ± 0.01bc	0.82 ± 0.06b	0.90 ± 0.04a	0.75 ± 0.03c

Lowercase letters in the same column indicate significant difference among treatments (*P* < 0.05).

**Table 3:** Functions of bacterial communities at tasseling stage (%).

Functions	S1N0	S1N1	S1N2	S1N3	S1N4	S0N2
1	40.61 ± 0.06b	40.61 ± 0.06b	40.55 ± 0.05b	40.22 ± 0.26c	40.44 ± 0.08b	40.94 ± 0.06a
2	9.34 ± 0.10a	9.31 ± 0.09a	9.34 ± 0.03a	9.15 ± 0.11b	9.33 ± 0.06a	9.42 ± 0.04a
3	8.18 ± 0.04b	8.19 ± 0.03b	8.21 ± 0.00ab	8.19 ± 0.02b	8.24 ± 0.03a	8.18 ± 0.03b
4	4.43 ± 0.04b	4.43 ± 0.03b	4.41 ± 0.00bc	4.39 ± 0.03bc	4.37 ± 0.02c	4.47 ± 0.02a
5	4.21 ± 0.03ab	4.20 ± 0.02ab	4.19 ± 0.01abc	4.18 ± 0.03bc	4.16 ± 0.02c	4.23 ± 0.02a
6	2.80 ± 0.06b	2.81 ± 0.04b	2.77 ± 0.01bc	2.71 ± 0.08c	2.70 ± 0.04c	2.90 ± 0.02a
7	2.81 ± 0.04bc	2.76 ± 0.02c	2.83 ± 0.03abc	2.89 ± 0.09a	2.88 ± 0.05ab	2.66 ± 0.01d
8	2.39 ± 0.02ab	2.38 ± 0.01ab	2.38 ± 0.01ab	2.37 ± 0.03ab	2.36 ± 0.02b	2.39 ± 0.02a
9	2.31 ± 0.01b	2.32 ± 0.01b	2.30 ± 0.00bc	2.29 ± 0.02c	2.29 ± 0.01c	2.35 ± 0.01a
10	2.34 ± 0.03ab	2.29 ± 0.02c	2.31 ± 0.01bc	2.36 ± 0.05a	2.32 ± 0.02bc	2.25 ± 0.02d
11	2.28 ± 0.02b	2.27 ± 0.03b	2.28 ± 0.01b	2.37 ± 0.06a	2.31 ± 0.02b	2.20 ± 0.01c
12	2.24 ± 0.02bc	2.24 ± 0.02bc	2.25 ± 0.01bc	2.26 ± 0.03ab	2.27 ± 0.01a	2.22 ± 0.01c
13	1.96 ± 0.05b	1.97 ± 0.01b	1.99 ± 0.02ab	2.05 ± 0.07a	2.04 ± 0.04a	1.90 ± 0.02c
14	1.56 ± 0.01bc	1.56 ± 0.00ab	1.55 ± 0.00cd	1.55 ± 0.01d	1.56 ± 0.00bcd	1.57 ± 0.01a
15	1.58 ± 0.01c	1.60 ± 0.01ab	1.60 ± 0.01abc	1.62 ± 0.02a	1.62 ± 0.01a	1.59 ± 0.02bc
16	1.37 ± 0.02b	1.37 ± 0.01b	1.36 ± 0.01bc	1.34 ± 0.03c	1.34 ± 0.01c	1.40 ± 0.01a
17	1.17 ± 0.01c	1.20 ± 0.01b	1.17 ± 0.01c	1.16 ± 0.02c	1.17 ± 0.01c	1.22 ± 0.02a
18	1.11 ± 0.01a	1.11 ± 0.00a	1.10 ± 0.01a	1.10 ± 0.01a	1.11 ± 0.00a	1.10 ± 0.01a
19	0.86 ± 0.02bc	0.88 ± 0.02b	0.87 ± 0.01bc	0.91 ± 0.02a	0.87 ± 0.02bc	0.85 ± 0.01c
20	0.79 ± 0.03b	0.76 ± 0.03b	0.77 ± 0.01b	0.85 ± 0.06a	0.77 ± 0.02b	0.70 ± 0.01c

Lowercase letters in the same column indicate significant difference among treatments (*P* < 0.05).

**Table 4:** Functions of bacterial communities at maturity stage (%).

Functions	S1N0	S1N1	S1N2	S1N3	S1N4	S0N2
1	40.86 ± 0.08ab	40.95 ± 0.12a	40.97 ± 0.19a	40.54 ± 0.09c	40.68 ± 0.24bc	40.96 ± 0.12a
2	9.29 ± 0.04ab	9.28 ± 0.09ab	9.41 ± 0.16a	9.26 ± 0.11ab	9.24 ± 0.07b	9.28 ± 0.04ab
3	8.12 ± 0.02abc	8.11 ± 0.01bc	8.16 ± 0.06ab	8.17 ± 0.04a	8.14 ± 0.02abc	8.10 ± 0.01c
4	4.50 ± 0.02ab	4.52 ± 0.00ab	4.49 ± 0.05abc	4.44 ± 0.04c	4.47 ± 0.03bc	4.54 ± 0.03a
5	4.26 ± 0.01ab	4.26 ± 0.01ab	4.24 ± 0.02b	4.21 ± 0.03c	4.23 ± 0.02bc	4.27 ± 0.01a
6	3.00 ± 0.05ab	3.05 ± 0.01a	2.97 ± 0.10ab	2.84 ± 0.08c	2.93 ± 0.09bc	3.08 ± 0.05a
7	2.66 ± 0.04bc	2.57 ± 0.03c	2.62 ± 0.10bc	2.82 ± 0.09a	2.73 ± 0.11ab	2.59 ± 0.05c
8	2.45 ± 0.03abc	2.48 ± 0.01ab	2.45 ± 0.04abc	2.41 ± 0.05c	2.44 ± 0.03bc	2.49 ± 0.01a
9	2.36 ± 0.01ab	2.37 ± 0.01a	2.36 ± 0.03ab	2.31 ± 0.01c	2.33 ± 0.03bc	2.37 ± 0.02a
10	2.28 ± 0.02abc	2.24 ± 0.04bc	2.23 ± 0.05c	2.34 ± 0.05a	2.30 ± 0.06ab	2.25 ± 0.02bc
11	2.22 ± 0.02ab	2.18 ± 0.02b	2.18 ± 0.05b	2.27 ± 0.02a	2.24 ± 0.07ab	2.17 ± 0.03b
12	2.21 ± 0.00c	2.22 ± 0.01bc	2.22 ± 0.01bc	2.25 ± 0.01a	2.23 ± 0.02ab	2.21 ± 0.01c
13	1.83 ± 0.04bc	1.81 ± 0.03c	1.85 ± 0.06bc	1.96 ± 0.05a	1.91 ± 0.06ab	1.78 ± 0.04c
14	1.58 ± 0.01abc	1.59 ± 0.00ab	1.58 ± 0.02abc	1.56 ± 0.01c	1.57 ± 0.01bc	1.59 ± 0.01a
15	1.52 ± 0.02ab	1.50 ± 0.02b	1.53 ± 0.01ab	1.55 ± 0.04a	1.52 ± 0.02ab	1.49 ± 0.01b
16	1.42 ± 0.01ab	1.43 ± 0.01a	1.42 ± 0.03ab	1.37 ± 0.02c	1.39 ± 0.03bc	1.44 ± 0.01a
17	1.21 ± 0.02ab	1.24 ± 0.02a	1.21 ± 0.04ab	1.17 ± 0.02c	1.19 ± 0.03bc	1.23 ± 0.02a
18	1.13 ± 0.02a	1.15 ± 0.03a	1.13 ± 0.01a	1.13 ± 0.03a	1.15 ± 0.00a	1.15 ± 0.00a
19	0.85 ± 0.01ab	0.85 ± 0.01ab	0.84 ± 0.02b	0.87 ± 0.02a	0.86 ± 0.01ab	0.85 ± 0.01b
20	0.80 ± 0.02ab	0.78 ± 0.05ab	0.74 ± 0.05b	0.83 ± 0.05a	0.83 ± 0.06a	0.78 ± 0.03ab

1, Global and overview maps; 2, Carbohydrate metabolism; 3, Amino acid metabolism; 4, Energy metabolism; 5, Metabolism of cofactors and vitamins; 6, Translation; 7, Membrane transport; 8, Replication and repair; 9, Nucleotide metabolism; 10, Cellular community-prokaryotes; 11, Signal transduction; 12, Lipid metabolism; 13, Xenobiotics biodegradation and metabolism; 14, Biosynthesis of other secondary metabolites; 15, Metabolism of other amino acids; 16, Folding, sorting and degradation; 17, Glycan biosynthesis and metabolism; 18, Metabolism of terpenoids and polyketides; 19, Cell growth and death; 20, Cell motility. Lowercase letters in the same column indicate significant difference among treatments (*P* <0.05).

### 3.7 Yield and yield components

According to Table 5, the ear width and ear length were significantly higher in S1N4 compared with S1N1, with the highest values recorded as 52.32 ± 2.06 cm and 22.54 ± 0.63 cm, respectively. There were no significant differences in ear width and ear length between S1N3 and S1N4, as well as between S1N2 and S0N2. The bald length in S1N3 was the smallest and differed significantly from S1N0 and S1N1 but did not differ significantly from S1N4. Except for S1N0, there were no significant differences in the number of kernels per row among the different treatments. The 100 grain weight followed the order of S1N4 > S1N2 > S1N3 > S0N2 > S1N1 > S1N0, but there were no significant differences among S1N2, S1N3, S1N4, and S0N2. The yields of S1N3 were 15597.85 ± 1477.17 kg/hm^2^, which was 12.80 and 4.18% higher than those of S1N1 and S0N2, respectively. The yields of S1N3 and S1N4 were the highest among all treatments, attributable to their superior ear width, ear length, and 100-grain weight. There were no significant differences in each index between S1N3 and S1N4.

**Table 5:** Yield and yield components.

Treatment	Ear diameter (cm)	Ear length (cm)	Bare tip length (cm)	The number of row per ear	The number of kernel per row	Hundred grain weight (g)	Yield (kg hm^−2^)
S1N0	43.84 ± 0.62c	18.03 ± 0.54c	2.51 ± 0.34a	16.07 ± 0.67a	28.19 ± 1.92b	28.94 ± 1.09c	7415.90 ± 350.27c
S1N1	50.41 ± 0.90b	21.39 ± 0.70b	1.44 ± 0.30b	16.60 ± 0.38a	39.07 ± 3.10a	37.20 ± 1.54b	13827.81 ± 1339.65b
S1N2	50.71 ± 1.50ab	21.98 ± 0.69ab	1.04 ± 0.47bc	16.80 ± 0.31a	40.21 ± 1.91a	40.10 ± 1.73a	15147.05 ± 1236.23ab
S1N3	52.12 ± 0.99a	21.76 ± 1.11ab	0.75 ± 0.36c	16.70 ± 0.30a	41.99 ± 2.23a	39.60 ± 1.68a	15597.85 ± 1477.17a
S1N4	52.32 ± 2.06a	22.54 ± 0.63a	0.87 ± 0.37c	16.87 ± 0.43a	41.38 ± 2.28a	40.87 ± 1.53a	16259.73 ± 925.50a
S0N2	50.94 ± 1.21ab	22.26 ± 0.74ab	0.95 ± 0.27c	16.55 ± 0.31a	40.56 ± 1.72a	39.11 ± 1.32a	14972.39 ± 1486.26ab

Lowercase letters in the same column indicate significant difference among treatments (*P* < 0.05).

## 4 Discussion

### 4.1 Effects of combined slow-release nitrogen fertilizer with urea on soil bacterial community structure under straw return conditions

Microorganisms play a crucial role in ecosystems, serving as the primary biological factor and facilitating the movement of materials and energy. Within the soil ecosystem, soil microorganisms are particularly significant as they contribute to the organic carbon conversion and retention and soil fertility development (Kušliene et al., 2014). Scientists are highly interested in the microbial composition of soil as it serves as a sensitive indicator of soil quality, regulating plant growth and soil nutrient balance while also reflecting changes in farmland and soil environment (Bu et al., 2020; Wu et al., 2020; Yan et al., 2020). In a study by Wolna-Maruwka et al. (2021), the main dominant phyla in maize soils were Actinobacteria, Proteobacteria, Firmicutes, Planctomycetes, Acidobacteria, Verrucomicrobia, Bacteroidetes, Chloroflexi, and Gemmatimonadetes. Similarly, Wierzchowski et al. (2021) observed that the main dominant phyla in maize cultivation soil were Verrucomicrobia, Proteobacteria, Planctomycetes, Gemmatimonadetes, Firmicutes, Chloroflexi, Bacteroidetes, Actinobacteria, and Acidobacteria. The dominant soil phyla in this study consisted mainly of Proteobacteria, Actinobacteriota, Acidobacteriota, Chloroflexi, Gemmatimonadota, Firmicutes, Bacteroidota, and Myxococcota. Furthermore, these dominant phyla were frequently found in other types of agricultural fields (Jiao et al., 2023; Khmelevtsova et al., 2023) and were considered the prevailing group in most agricultural soils (Eo and Kim, 2022; Kodadinne-Narayana et al., 2022).

This study found that the bacterial phyla composition remained consistent between the full maize straw return and straw removal. However, there was a significant change in bacterial abundance (Figure 2). Straw returning increased the relative abundances of Proteobacteria and Bacteroidota while decreasing the relative abundances of Acidobacteriota and Planctomycetota. In a similar study, Yang et al. (2019) investigated the effects of wheat straw returning on soil bacterial communities in wheat–soybean rotation systems over a 3-year experiment. They observed that wheat straw returning increased the relative abundance of Proteobacteria and decreased the relative abundance of Acidobacteria (Eo and Kim, 2022). Another study by Dang et al. (2021) demonstrated that straw addition promoted the growth of Proteobacteria, Actinobacteriota, and Bacteroidota while inhibiting the growth of Acidobacteriota and Nitrospirota. Chen et al. (2019) also found that Actinobacteriota, Proteobacteria, and Gemmatimonadota were significantly enriched with the application of maize straw and biochar, but the relative abundance of Acidobacteriota decreased. The conclusions of this study align with the aforementioned research findings.

The addition of extra nutrients during the straw returning process is crucial for determining the growth of the soil microbial population. Organic matter, which serves as an important carbon source for microbial reproduction (Hao et al., 2019), is a primary factor contributing to changes in microbial abundance. Proteobacteria, the largest bacteria phylum in the soil, exhibit a strong survival ability and can quickly adapt to various complex and changing environments with low living condition requirements (Rampelotto et al., 2013; Liu et al., 2014). Due to its ubiquity, high abundance, species richness, and genetic diversity (Chen et al., 2017), Proteobacteria play a vital role in soil nutrient cycling (Ren et al., 2018), especially in organic-rich environments. During the jointing stage, there was a significant increase in the presence of Proteobacteria in S1N2, S1N3, and S1N4, with increases of 9.48, 25.62, and 42.37%, respectively, compared with S0N2. Similarly, at the tasseling extraction stage, there was a remarkable increase in the abundance of Proteobacteria in S1N2, S1N3, and S1N4, with increases of 29.21, 55.79, and 34.69%, respectively, compared with S0N2. At the maturity stage, the abundance of Proteobacteria increased by 6.42, 39.77, and 25.90%, respectively. Bacteroidota members exhibited symbiotic properties and were highly enriched in soils with high carbon supply (Bai et al., 2020). Biochar application had a significant impact on the relative abundances of Proteobacteria and Bacteroidota during wheat’s flowering stage (Yao et al., 2023). In this study, there was a notable difference in the abundance of Bacteroidota between maize’s tasseling and maturity stages. When comparing S1N2, S1N3, and S1N4 with S0N2, the abundance of Bacteroidota increased by 65.93, 127.41, and 118.14%, respectively, during the tasseling stage. Similarly, during the maturity stage, the abundance of Bacteroidota increased by 37.58, 117.88, and 61.59%, respectively. Acidobacteria are predominantly found in oligotrophic groups. Although the soil nutrient status improved after straw return, it did not reach eutrophic levels. However, compared with the straw removal treatment, the soil environment improved significantly, promoting the growth of other bacterial phyla while inhibiting the growth of Acidobacteria (Feng et al., 2021). In this study, the abundance of Acidobacteria decreased by 32.18, 51.89, and 36.60% at the jointing stage when comparing S1N2, S1N3, and S1N4 with S0N2, respectively. Similarly, at the tasseling stage, the abundance of Acidobacteria decreased by 19.47, 51.75, and 42.53%, respectively.

The analysis showed that when straw was returned, a low slow-release fertilizer application rate (S1N2) had minimal impact on the relative abundance at the phylum level. However, the middle and high application rates of slow-release fertilizer (S1N3 and S1N4) significantly altered the relative abundances of Proteobacteria, Acidobacteriota, Meheylpmirabilota, Planctomycetota, Bacteroidota, and other phyla. Deep application of slow-release fertilizer improved the structure, distribution, and diversity of the microbial community in the rice rhizosphere compared with broadcasting (Chen et al., 2022). Wu et al. (2019) found that slow-release fertilizer application did not significantly alter the structure of the microbial community in acidic soil compared with conventional fertilization. The conclusions regarding the impact of slow-release fertilizer on the soil bacterial community are not unified, possibly due to various factors such as soil texture, physical and chemical properties, geographical location, and agricultural management methods. Therefore, there is a need to enhance our understanding of how slow-release fertilizer affects soil microbial communities under different agricultural management practices.

When the regional environment changes during the crop growth process, it can have varying effects on the growth of microorganisms. Some microorganisms may be promoted, while others may be inhibited. In the case of straw returning to a field, it alters the soil environment in the region and enhances the soil organic matter content. This change provides a more favorable habitat for soil bacteria, leading to an increase in the abundance of eutrophic bacteria and a decrease in the abundance of oligotrophic bacteria.

### 4.2 Relationship between the straw decomposition rate and the bacterial phylum

Straw decomposition is a process of organic carbon mineralization and nutrient release regulated by soil microorganisms. In maize straw decomposition, Actinobacteria, Firmicutes, and Proteobacteria play crucial roles (Fan et al., 2014). This study identified six phyla that are key players in straw decomposition. Among these, Proteobacteria, Actinobacteriota, Myxococcota, and Bacteroidota showed a positive correlation with straw decomposition, while Acidobacteriota and Chloroflexi showed a negative correlation. Notably, Proteobacteria and Bacteroidota had the greatest influence. Previous studies have highlighted that Actinobacteriota produces active enzymes that effectively degrade organic carbon, thereby accelerating the decomposition of straw and other organic matter. The abundance of Actinobacteriota is directly proportional to the straw decomposition ability of microorganisms (Trivedi et al., 2013; Fan et al., 2014). In this study, Actinobacteriota was the second most abundant phylum after Proteobacteria.

Chloroflexi, a widely studied oligotrophic bacteria, is known for its ability to degrade macromolecular organic matter into smaller molecules. It is highly sensitive to soil pH and thrives under neutral conditions (Xu et al., 2019). However, in this study, Chloroflexi showed a negative correlation with straw decomposition, possibly due to the alkaline soil in the study area, which was not conducive to its growth. Another important player in organic matter decomposition is Firmicutes, which promotes cellulose degradation and contributes to the carbon cycle (Fan et al., 2014; Zhao et al., 2019). Interestingly, in this study, there was a negative correlation between Firmicutes and straw return to a field (Figure 6), although the difference was insignificant. These findings suggest that regional variations in soil physical and chemical properties can greatly influence the microbial community composition (Zhang et al., 2014). Furthermore, the evolution of microbial composition during straw decomposition may also vary across different regions.

The straw decomposition process can be divided into fast and slow stages. This study found that the maize decomposition rate was faster before the tasseling stage than afterward. This finding is consistent with a previous study (Li et al., 2017), which observed that straw decomposition was fast in the early stage and slow in the late stage.

Bastian et al. (2009) and Sun et al. (2013) observed that the microbial communities involved in straw decomposition undergo a succession of nutrient status, transitioning from saturation to depletion. Nutrient-rich bacteria primarily regulate the decomposition process in the early stage. In contrast, the relative abundance of nutrient-poor bacteria increases as straw quality declines, dominating the decomposition process in the middle and late stages. Eutrophic bacteria such as Bacteroidetes, Firmicutes, and Proteobacteria are responsible for decomposing the nutrient-rich and easily degradable fractions of straw in the early stage (Fierer et al., 2007). On the other hand, nutrient-poor bacteria such as Chloroflexi, Saccharibacteria, and Acidobacteria dominate the degradation of nutrient-poor and difficult-to-break-down fractions in the middle and late stages of straw decomposition (Fierer et al., 2007). In the case of maize straw decomposition, Fan et al. (2014) found that Proteobacteria play a crucial role in the pre-decomposition stage, while Acidobacteria are significant in the post-decomposition stage. In our study, we observed that different bacterial phyla dominate straw decomposition at different stages. Specifically, Proteobacteria, Bacteroidota, Actinobacteriota, and Myxococcota were the dominant phyla during the pre-reproductive stage, while Proteobacteria and Bacteroidota were dominant during the late reproductive stage. Interestingly, Acidobacteria showed a negative correlation with straw decomposition in both the pre-reproductive and post-reproductive stages. This might be attributable to the prolonged release of slow-release nitrogen fertilizer used in our experiment, which fulfilled the nutrient requirements of microorganisms at all stages, particularly during the late reproductive stage, resulting in a eutrophic soil environment that inhibited Acidobacteria. The changes in soil carbon and nitrogen content are likely the main factors influencing bacterial community evolution during straw decomposition.

## 5 Conclusion

Under a full straw returning system, the combined use of slow-release nitrogen fertilizer and urea had significant effects on the soil bacterial community and structure, straw decomposition rate, and maize yield. Straw returning led to a significant increase in the relative abundances of Proteobacteria and Bacteroidota while decreasing the relative abundances of Acidobacteriota and Planctomycetota. The low application rate of slow-release fertilizer (S1N2) had minimal impact on the relative abundance of phyla, whereas the middle and high application rates (S1N3 and S1N4) significantly altered the relative abundance of bacterial phyla. The cumulative straw decomposition rate in S1N3 and S1N4 was significantly higher (*P* < 0.05) compared with other treatments. The yield of S1N3 was 15597.85 ± 1477.17 kg/hm^2^, representing 12.80 and 4.18% increases over S1N1 and S0N2, respectively. There was no significant difference in soil bacterial community, structure, straw decomposition rate, and maize yield between S1N3 and S1N4. Therefore, we can conclude that considering input and expenditure, the optimal application proportion of slow-release nitrogen fertilizer and urea is 60%:40%.

## Data availability statement

The original contributions presented in the study are included in the article/supplementary material, further inquiries can be directed to the corresponding author.

## Author contributions

LY: Supervision, Writing – original draft. DL: Data curation, Writing – review & editing. YZ: Formal analysis, Writing – review & editing. YW: Formal analysis, Writing – review & editing. QY: Investigation, Writing – review & editing. KY: Funding acquisition, Project administration, Writing – review & editing.

## Footnotes

^1^ https://cloud.majorbio.com

^2^ http://huttenhower.sph.harvard.edu/LEfSe
